# The dual roles of cytokines in Alzheimer’s disease: update on interleukins, TNF-α, TGF-β and IFN-γ

**DOI:** 10.1186/s40035-016-0054-4

**Published:** 2016-04-05

**Authors:** Cong Zheng, Xin-Wen Zhou, Jian-Zhi Wang

**Affiliations:** Department of Pathophysiology, School of Basic Medicine and the Collaborative Innovation Center for Brain Science, Key Laboratory of Ministry of Education of China for Neurological Disorders, Tongji Medical College, Huazhong University of Science and Technology, Wuhan, 430030 China; Co-innovation Center of Neuroregeneration, Nantong, 226000 China

**Keywords:** Alzheimer’s disease, Cytokines, Interleukins, TNF-α, TGF-β, IFN-γ

## Abstract

Alzheimer’s disease (AD) is one of the most common neurodegenerative disorders in the elderly. Although the mechanisms underlying AD neurodegeneration are not fully understood, it is well recognized that inflammation plays a crucial role in the initiation and/or deterioration of AD neurodegeneration. Increasing evidence suggests that different cytokines, including interleukins, TNF-α, TGF-β and IFN-γ, are actively participated in AD pathogenesis and may serve as diagnostic or therapeutic targets for AD neurodegeneration. Here, we review the progress in understanding the important role that these cytokines or neuroinflammation has played in AD etiology and pathogenesis.

## Background

Dementia has become a global challenge for public health. Currently, over 40 million people worldwide live with this condition and this number would double by 2030 and more than triple by 2050 [1]. Alzheimer’s disease (AD) is the most prevalent cause of dementia, characterized by progressive cognitive and functional impairments and as well as memory deterioration. Although much effort has been made in the past several decades to uncover the mechanism of AD pathogenesis and to further translate these findings into the clinic, there are still no mechanism-based treatments approved for this devastating disease and the current therapies only provide transient symptomatic release.

The two most well-known pathological hallmarks of AD are extracellular amyloid plaques comprised of aggregated Aβ, and intracellular neurofibrillary tangles (NFTs) generated by hyperphosphorylated microtubule-associated protein tau. Increasing evidence indicates that neuroinflammation can act as an independent factor at very early stage of AD, where the immune-related genes and cytokines are the key participants.

Cytokines are a heterogeneous group of proteins with molecular weights ranging from 8 to 40 kDa. These multifunctional molecules can be synthesized by nearly all nucleated cells and generally act locally in a paracrine or autocrine manner. Many of them are referred to as interleukins (ILs), indicating that they are secreted by and act on leukocytes. Other important types of cytokines, such as tumor necrosis factors (TNFs), interferons (IFNs) and transforming growth factors (TGFs) that can cause cell death, activate natural killer cells and macrophages, and induce phenotypic transformation and act as a negative autocrine growth factor, respectively. Another member of the big cytokine family is the chemokines, which can attract and activate leukocytes. In view of their relatively exclusive functions, chemokines are usually discussed separately.

As cytokines are rapidly changed in response to infections or trauma, they have been classified as either “pro-inflammatory” or “anti-inflammatory”. The balance between the two types of cytokines guarantees immediate elimination of the invading pathogens and the timely withdraw of excessive reaction, which is the key to preventing many diseases including the neurodegenerative diseases. The expression of cytokine receptors is temporally and spatially regulated in the central nervous system (CNS) [2], and they are closely involved in cell proliferation, gliogenesis, neurogenesis, cell migration, apoptosis, and synaptic release of neurotransmitters [3, 4].

Cytokines have attracted much attention towards their exact roles in different stages of AD and the possibility for therapeutics. However, cytokines levels detected in AD patients were inconsistent among different research groups, while regulating the expression of cytokines in AD animal models yielded unexpected results as well. Here, we will focus on the most extensively studied cytokines, including ILs, TNF-α, TGF-β and IFN-γ, looking for the commonness, reasoning the disagreement among recent studies and give suggestions about how to translate these precious findings from the laboratories to the clinic in AD.

## Evidence from AD patients

The postmortem analysis of the AD brains has provided pioneering evidence for involvement of inflammation in AD pathology. IL-1β [5], IL-6 [6] and TGF-β [7] and many other cytokines have been found to accumulate around the amyloid plaques in the brain of AD patients, which led to numerous studies investigating the levels of pro-inflammatory and anti-inflammatory cytokines in the cerebral spinal fluid (CSF) or serum of patients with mild cognitive impairment (MCI) or AD. Although results are inconclusive, there appears a trend that pro-inflammatory (IL-1β, IL-6, TNF-α) and anti-inflammatory cytokines (IL-1 receptor antagonist (IL-1ra), IL-10) are both elevated in the CSF and plasma of AD patients [8]. The alterations of cytokine levels reflect the disturbance of immune system in AD, however, the evidence from the body fluid is insufficient to decide whether these changes are a initiating or secondary event of the disease, thus more approaches should be adopted to illustrate a more reliable picture for the role of cytokines.

Although the established genetic causes such as gene mutations encoding amyloid precursor protein (*APP*), presenelin 1 (*PSEN1*) and *PSEN2* are only dominant to a minority of familial type of AD, these risk genes have deepened our understanding of AD mechanisms in many aspects. For instance, the heterozygous rare variants in gene coding triggering receptor expressed on myeloid cells 2 (TREM2) increases risk of AD with an unfavorable inflammatory condition for Aβ clearance [9], thus shedding a light on the possible initiating role of inflammation in AD pathogenesis.

To date, at least 23 cytokine polymorphisms involving 13 types of cytokines have been identified to be associated with AD. Based on the following three conditions (1) having polymorphisms that are significantly associated with AD, (2) having corresponding genotype/phenotype data, and (3) having previous records of the changed levels in AD patients, these cytokines can be divided into five groups as follows: (i) Cytokines like IL-1β, IL-6, IL-18 and TNF-α have the above three conditions. (ii) Cytokines like IL-4, IL-12, IL-23 and IFN-γ have the first two conditions but have no level change or the related data in AD, demanding new strategies to measure the cytokine level in AD patients, especially in those with the polymorphisms. (iii) Cytokines like IL-10 have conditions 1 and 3, calling for future studies. (iv) Cytokines like IL-1ra and TGF-β only meet condition 3 but have numerous evidence from both in vivo and in vitro studies, indicating that the genetic factor may not be crucial for their actions in AD or need further studies. (v) Cytokines like IL-16, IL-15 and IL-17 that either only have condition 1 or lack all three conditions still needs more evidence to confirm their involvement in AD.

Although many studies have discussed the polymorphism-related cytokine level changes, the data are mostly referenced from other research fields than AD, such as cancer. The direct evidence for the cytokine levels in different populations is also not convincing enough to draw a definite conclusion.

In the subgroup meta-analysis of cytokine polymorphisms, many grouping factors could decrease heterogeneity and improve significance, such as races [10–14], apolipoprotein E (ApoE) *ε4* allele and time of AD onset. As for races, it is rare to find significance in Asian and Caucasian populations, and in more extreme cases, a polymorphism indicates higher risk of AD in a population while shows lower risk in the other [12, 13]. This may be a result of the different frequencies of the polymorphism between different races and interplay of the variant with other unknown race-specific genes, or even with the environment. Of course, the influence from limited sample size of certain population cannot be excluded [15]. ApoE *ε4* allele, the widely recognized late-onset AD triggering factor, is associated with at least 5 cytokine polymorphisms [16–20], indicating a potential synergistic interaction between them. ApoE *ε4* can modify AD risk in patients with diabetes or cardiovascular disease, which could be attributable to related hyperlipidemia and hypercholesterolemia. ApoE4 could also independently cause neurovascular dysfunction through triggering inflammatory cascades [21]. Thus, it would be necessary to know whether cytokines play the initiating or secondary role in the interaction with ApoE. In addition, although few studies clarified the time of AD onset in their samples, it seems from the present studies the alteration cytokine levels have more influence on the late-onset AD (LOAD). As many cytokines have a close interaction with ApoE, whether this potential synergistic effect is the sole reason to the onset-time association deserves further investigation.

When single polymorphism of a cytokine does not always guarantee significance, the haplotype of one or more different types of cytokines may show associations with AD [20, 22, 23]. There is also a positive and linear relation between the numbers of the pro-inflammatory cytokine polymorphisms and AD risk [24], which suggests that their corresponding proteins might interact with each other in a cumulative manner [25].

Compared with the widely recognized genetic risk factors like TREM2 or CD33 [26], the genetic evidence from cytokines may be insufficient to prove that cytokines levels imbalance alone is able to trigger AD. However, a polymorphism of IFN-γ is associated with fast progressing AD makes it certain that cytokines could play an active role in exacerbating the AD course [27]. Together, the cytokine polymorphisms may not markedly assist in predicting AD risk, but they have irreplaceable value in identifying pathways involved in the disorder and potential drug targets. The relationship between cytokines with races and ApoE and some of the AD-related cytokine polymorphisms are summarized in Table 1.

**Table 1:** Cytokine polymorphisms and levels in serum or CSF

Cytokines^a^		Levels^b^				Polymorphisms/haplotypes		Corresponding cytokine expression^c^	Results^d^	Methods	Ref.
		MCI		AD							
		Plasma/serum	CSF	Plasma/serum	CSF						
IL-1 family [144]	IL-1α	=	\	= or ↓	\	-889 C/T (rs1800587)		T: ↑	* ↑ in EOAD	Meta-analysis	[145]
									No * in LOAD.		
									* ↑ in Caucasians	Meta-analysis	[14]
									No * in Asians.		
	IL-1β	↑	=	↑or =	= or ↑	-511 C/T (rs16944)		?	no*	Meta-analysis	[15]
						-31 T/C (rs1143627)		C: ?	*↓ in Italians	Case–control	[25]
						−511C/−31 T/IL1RN2		?	*↓ in elderly group of Brazilians	Case–control	[23]
						−511C/−31C/IL1RN1		?	* ↑ in Brazilians	Case–control	
						+3953 C/T (rs1143634)		T: ↑	* ↑ in non-Asians	Meta-analysis	[15], [14]
						Exon 5 E1/E2		\	No* in Taiwan population	Case–control	[146]
	IL-1ra	\	\	-	↓ [139]	Intron 2 I/II/IV		\			
	IL-18 [71]	=	\	= or ↑	↑	-607 A/C (rs1946518)		C: ↑	* ↑ in LOAD of Han Chinese.	Case–control	[20]
									*↑↑ in ApoE ε4 carrier.		
						-137 C/G (rs187238)		G: ↑			
						-607 C or -137 G		↑	* ↑ in LOAD of Han Chinese.		
	IL-33 [147]	\	\	\	\	rs11792633 C/T		T: ↑	*↓ in non-ApoE ε4 carrier in both Caucasians and Han Chinese.	Case–control	[19],[148]
	IL-4 [149]	\	\	\	\	-1098 T/G (rs2243248)		G: Possibly ↓	*↑ in Han Chinese	Case–control	[150]
								T: Possibly ↓	*↑ in Caucasians		[151]
						-590 C/T (rs2243250)		C: ↓	*↑ in Han Chinese and Caucasians		[150], [151]
IL-6 family	IL-6 [152, 153]	=	\	↑or =	= or ↑	-174 G/C (rs1800795)		C: ↓	*↓ in Asians, No* in Caucasians.	Meta-analysis	[10].
									*↓ in Italians.	Case–control	[25]
						-572 C/G (rs1800796)		C: ?	*↑ in ApoE ε4 carriers.	Case–control	[16]
	IL-11 [154]	=	\	= or ↑	↑	\		\	\	\	\
	IL-10 [155, 156]	=	↑	= or ↑	=	-1082 A/G (rs1800896)		A: ?	*↑ in Caucasians, No* in Asians.	Meta- analysis	[11]
								G: ↑	↓ in Caucasians	Meta- analysis	[22]
						-819 T/C (rs1800871)		?	no *		
						-592 A/C (rs1800872)					
						-1082G/-819C/-592C		?	↓		
IL-12 family [157]	IL-12A	=	\	=	=	rs2243115 T/G		G: ↓	*↓ in LOAD in ApoE ε4 carrier of Northern Han Chinese	Case–control	[17]
						rs568408 G/A		A: ↓	*↓ in LOAD of Northern Han Chinese		
	IL-12B	=	\	=	=	rs3212227 A/C		C: ↓	*↓ in LOAD of Northern Han Chinese		
	IL-23	\	\	\	\	rs10889677 A/C (L-23R)		C: ↓	*↓ risk in Northern Han Chinese.	Case–control	[18]
						rs1884444 T/G (IL-23R)		G: ↓	*↑ in ApoE ε4 carrier of Northern Han Chinese.	Case–control	
	IL-15 [158]	\	\	=	=[159]	\		\	\	\	\
	IL-16 [160]	\	\	\	\	rs4072111 C/T		T: ?	*↓ in LOAD of Iranians.	Case–control	[161]
	IL-17 [162]	\	\	\	\	\		\	\	\	\
	TNF-α [163]	= or ↑	↓	= or ↑ or ↓	= or ↑ or ↓	-308 G/A (rs1800629)		A: ↑	*↑ in East Asian	Meta-analysis	[12], [13]
									*↓ in Northern European population.		
									No* in Italians	Case–control	[25]
	TGF-β [164]	\	↓	↑ or ↓ or =	↑ or ↓ or =	\		\	\	\	\
	IFN-γ [165]	\	\	=	=	-874 T/A (rs62559044)		A: ↓	*↑ in fast progressing AD	Case–control	[27]

*Abbreviation*: *IL-1 ra* IL-1 receptor antagonist, *EOAD* early-onset Alzheimer’s disease, *LOAD* late-onset Alzheimer’s disease

^a^Each cytokine or cytokine family was supplemented with a latest review for detailed information of physiological parameters

^b^↑: up-regulated, ↓: down-regulated, =: no change, \: no data. Unless otherwise noted, all the data of cytokine levels is from Brosseron et al. 2014 [8]

^c^↑: enhance the cytokine expression, ↓: attenuate the cytokine expression, ?: unknown yet

^d^*: significant, ↑: higher risk of AD onset, ↓: lower risk of AD onset

## Cytokines related to AD-like Aβ abnormalities

As one of the most well-known hallmarks of AD, Aβ is actively involved in the neuroinflammation. It is believed that Aβ has a predominant role in launching the detrimental self-exaggerated inflammation process that is responsible for the disease progression. The Aβ peptide is derived from amyloid precursor protein (APP) by sequential cleavages of two membrane-bound proteases. Aβ of different length, especially Aβ_1-42_ then form soluble oligomers and fibrils, the latter is the major component of extracellular amyloid plaques. Soluble Aβ can be degraded by various extracellular proteases, while fibrillary Aβ is phagocytosed by microglia, the resident phagocytes of CNS, then enter the endolysosomal pathway [28]. Astrocytes are also capable of degrading Aβ, primarily the cerebrovascular Aβ [29]. The dysregulation of Aβ clearance process resulted from the skewing of microglia or astrocytes to pro-inflammatory state, characterized by elevated levels of pro-inflammatory cytokines and compromised ability in Aβ clearance, will lead to Aβ accumulation and a sustained immune activation.

Several environmental factors, including diabetes, obesity, aging that are associated with immune disturbance could trigger the phenotype transformation of glial cells [28] through either direct modulation of the relevant mediators [30] or epigenetic modification [31]. Then, elicited by a self-propagating circle through the interaction between Aβ and pro-inflammatory cytokines [32–34], the chronic inflammation state is ultimately independent of the primary stimulus, which is a possible explanation to the failure of anti-amyloid treatment strategies in late stage of AD [35].

Several anti-TNF-α biologic medications have rescued Aβ deposition, behavioral impairments and inflammation in AD animal models [36–39], suggesting that TNF-α is a detrimental factor in AD course and can serve as a reliable AD target. However, hippocampal expression of TNF-α in APP transgenic mice at early stage induced robust glial activation that attenuate Aβ plaques without altering the APP levels [40]. Although there was only a suspicious infiltration of peripheral immune cells, increased major histocompatibility complex class II (MHC-II) cells were detected in the TNF-α expressing mice, indicating an enhanced antigen-presenting efficiency and more frequent communication with infiltrating T cells, which may facilitate Aβ removal.

Several studies indicate that overexpression of IL-1β in APP/PS1 mice reduces Aβ plaque accompanied by an activated population of microglia with greater phagocytosis [41, 42]. It is proposed that this group of microglia might be endogenous Arg-1+ M2a phenotype induced by Th2 cytokines, such as IL-4, secreted by a group of cells recruited to the Aβ plaques during the sustained IL-1β neuroinflammation [42]. The mice deficient in IL-1R had lower recruitment of microglia to amyloid plaques, implying that IL-1β can mediate microglial chemotaxis [43]. Moreover, IL-4 can down-regulate TNF-α and up-regulate MHC-II, insulin-like growth factor (IGF)-1 and CD36 in microglia [44], and thus not only decrease the neurotoxicity but also promote the ability of presenting antigen to T cells [45]. Similar results were also seen in IL-6 [46]. These studies indicate that overexpressing pro-inflmmatory cytokines in CNS may generate Aβ-clearance-promoting effect with a peripheral responses involved. However, it is noteworthy that none of these studies have relevant behavioral results (see Table 2), thus we cannot assess the overall result of this type of cytokine modulation. It is reported that chronic neuronal TNF-α expression in 3xTg AD mice led to large amount of neuronal death [47]. Whether the enhanced local inflammation and direct neurotoxicity or periphery-mediated Aβ reduction has larger impact on the cognitive performance needs further studies. Moreover, as the expressions of human APP or tau in AD animal models are driven by various unnatural transgene promoters, the possibility that some anti-cytokine molecules may act through interacting with these regulatory elements cannot be ruled out [37]. Therefore, a critical verification with alternative AD models is needed.

**Table 2:** Methods and results from in vivo studies of cytokines

Cytokines^a^	Animals	Main AD-like Pathology and initiating time	Cytokines Expression System		Expression Duration	Results^b^		Ref.
			Delivery Method	Administration Routes		Immuno-histochemistry	Behaviors	
**IL-1β**	3xTg AD mice (9 months old)	Aβ plaque: 6 mo. Tau: 12 -15 mo.	anti-IL-1R blocking antibody	Peritoneal Injection	every 8-9 days for 6 months	Aβ deposition ↓; Tau phosphorylation ↓	Cognition ↑	[83]
	Rats adult	-	IL-1β injections	Cerebral ventricles	1 d	TNF-α, IL-10 ↑	No significance	[34]
					8 d	TNF-α, IL-1β ↑ IL-10 ↓; APP mRNA ↑	Memory ↓	
	3xTg AD mice (8 months old)	Aβ plaque: 6 mo. Tau:12 -15 mo.	IL-1β-XAT cassette	Subiculum	1 and 3 mo.	Aβ deposition ↓; Tau phosphorylation ↑	\	[41]
	APPswe/PSEN1dE9 mice (8 months old)	Aβ plaque: 6 mo.	rAAV2-IL-1β	Hippocampi	1 mo.	Aβ deposition ↓	\	[42]
IL-6	TgCRND8 mice (0 -12 h old (P0)/36 -48 h old (P2))	Early Aβ plaque: 3mo. Dense-cored plaques: 5 mo.	rAAV2/1-IL-6	Cerebral ventricles	5 mo.	Aβ deposition ↓	\	[46]
	TgCRND8 mice (4 mo.)		rAAV2/1-IL-6	Hippocampi	1-1.5 mo.	Aβ deposition ↓	\	
	Tg2576 mice (P0)	Numerous Aβ plaques:11-13 mo.	rAAV2/1-IL-6	Hippocampi	3 mo.	Aβ deposition ↓	\	
**IL-4**	Tg2576 + PS1 mice (3 months old)	Aβ plaques: 6 mo.	rAAV2/1-IL-4	Hippocampi	5 mo.	Aβ↓; Gliosis ↓; Neurogenesis ↑	Spatial learning ↑	[54]
	TgCRND8 mice (4 months old)	Early Aβ plaque: 3 mo.	rAAV2/1-IL-4	Hippocampi	1.5 mo.	Aβ↑; Gliosis ↑	\	[55]
	APPswe/PSEN1dE9 mice (3 months old)	Aβ plaque: 6 mo.	rAAV2/1-IL-4	Frontal cortex, Hippocampi	43 d.	Aβ↓ with no significance; Enhanced M2a phenotype of microglia	\	[56]
**IL-10**	APPswe/PSEN1dE9 mice (3 months old)	Aβ plaques: 6 mo.	rAAV2/1-IL-10	Hippocampi	5 mo.	Aβ =; Gliosis ↓; Neurogenesis ↑.	Spatial learning ↑	[52]
	TgCRND8 mice (P0/P2)	Early Aβ plaque: 3mo.	rAAV2/1-IL-10	Cerebral ventricles	6 mo.	Aβ deposition ↑	Cognition ↓	[51]
	Tg2576 mice (8 months old)	Numerous Aβ plaques: 11-13 mo.	rAAV2/1-IL-10	Hippocampi	5 mo.	Aβ deposition ↑	Cognition ↓	
	APPswe/PSEN1dE9 mice	Aβ plaque: 6 mo.	Bred with IL-10 KO mice	The whole body	12-13 mo.	Aβ deposition ↓	Cognition ↑	[53]
IL-12/IL-23	APPswe/PSEN1dE9 mice	Aβ plaque: 6 mo.	Bred with p40 (IL-12 and IL-23 shared) KO, p35 (IL-12) KO or p19 (IL-23) KO mice	The whole body	4 mo.	Aβ deposition ↓ (especially with p40 KO)	Cognition ↑	[65]
	Senescence accelerated mouse (SAMP8) mice (6 months old)	Accelerated aging.	siRNA KO of p40	Dorsal third ventricle	1 mo.	Aβ deposition ↓	Cognition ↑	[66]
**TNF-α**	TgCRND8 mice (4 months old)	Early Aβ plaque: 3 mo.	rAAV2/1-TNF-α	Hippocampi	1.5 mo.	Aβ deposition ↓	\	[40]
	3xTg AD mice (10, 17 months old)	Aβ plaque: 6 mo. Tau: 12 -15 mo.	TNF-α-lowering agent (3,6'-dithiothalidomide)	Peritoneal Injection	1.5 mo.	APP, Aβ peptide and Aβ deposition ↓; Tau phosphorylation ↓	Cognition ↑	[37]
	3xTg AD mice (6 months old)		TNF-α-lowering agent (IDT)	Oral administration	10 mo.	Fibrillar Aβ↓; PHF-tau ↓	Cognition ↑	[39]
TGF-β	hAPP J9 line mice	Aβ plaques :5-7 mo.	Bred with transgenic expressing astrocytes-induced TGF-β1 mice	Brain	12-15 mo.	Aβ deposition ↓; Perivascular Aβ deposition ↑	\	[57]
	Transgenic mice with inducible neuron-specific expression of TGF-β1 (3 months old)	-	The heterologous tTA system	Neocortex, hippocampi, striatum	54 d	Perivascular Aβ deposition ↑	\	[62]
					24 d	Death of neurons induced by 3-nitropropionic acid ↓	\	
	SD rats with Aβ1-42 injection in bilateral hippocampus	Aβ	TGF-β1 injection 7 d after Aβ injection	Left cerebral ventricles	3d	APP ↓	Cognition ↑	[166]
	SD rats with Aβ1-42 injection in bilateral hippocampus	Aβ	TGF-β1 administration1h prior to Aβ injection	Cerebral ventricles	7 d	APP ↓; PP2A ↑; TNF-α, IL-1β, iNOS, IFN-γ, IL-2, IL-17 and IL-22 ↓.	Cognition ↑	[58]
			TGF-β1 administration7 d after Aβ injection	Nares	7 d	PP2A ↑; IL-1β, iNOS, IFN-γ, IL-2 and IL-17 ↓.	Cognition ↑	
**IFN-γ**	APP Tg J20 mice	Aβ plaques : 5-7 mo.	Bred with Tg SJL mice expressing IFN-γ	The whole body	9 mo.	Oligodendrogenesis ↓	\	[167]
	3xTg AD mice (2 months old)	Aβ plaque: 6 mo. Tau:12 -15 mo.	rAAV2/1- IFN-γ	Hippocampi	10 mo.	Aβ deposition ↑; Tau phosphorylation ↓	\	[168]
	TgCRND8 mice P2	Early Aβ plaque: 3mo. Dense-cored plaques: 5 mo.	rAAV2/1- IFN-γ	Cerebral ventricles	5 mo.	Aβ deposition ↓; Gliosis ↑; Complement expression ↑; Peripheral monocytes infiltration ↑	\	[63]
	TgCRND8 mice (4 months old)			Hippocampi	1.5 mo.			
	JNPL3 mice (P2), rTg4510 mice (P2)	Tau:4 mo.	rAAV2/1- IFN-γ	Cerebral ventricles	3 mo.	Soluble tau phosphorylation ↑	\	[87]

*Abbreviation*: *PHF-tau* Paired helical filament tau, *KO* knockout

^a^Cytokines with controversial results are in bold.

^b^↑: increase or improve, ↓: decrease or exacerbate, =: no change, \: no data

For more detailed information for the model animals mentioned above, please refer to http://www.alzforum.org/research-models [169]

On the other hand, the typical anti-inflammatory cytokines such as IL-4 and IL-10 suppress the inflammation through inhibiting the secretion of IL-1β, IL-6, TNF-α by microglia [48–50] in vitro. In contrast to IL-4 that triggers M2a activation state associated with development of an anti-inflammatory environment and enhanced phagocytosis, IL-10 drives M2c polarization that is associated with deactivation of microglia. Overexpressing IL-10 in several AD animal models weakened the phagocytosis of soluble Aβ by microglia and exacerbeted Aβ deposits with cognitive impairment [51–53]. Although inconsistent outcomes do exist, a recent study using IL-10 knockout mice supports the benefit of IL-10 removal. Considering that the IL-10 level increased in AD patients [53], it appears that the imbalance of pro- and anti- inflammatory activity co-exist in AD. Whether there is a corresponding, sequential transfer of microglia from M1 to M2c or mixed phenotype is unclear. It is also interesting to know whether this kind of transformation indicate exacerbation of the disease and “a point of no return” of the disease. As the previous in vivo studies of IL-10 all gave intervention before the formation of typical AD pathology (see Table 2), more data of the IL-10 impact on the late stage of AD is required.

The in vivo IL-4 studies generated more controversial results: One shows that overexpression of IL-4 in pre-deposition phase of AD animal models resulted in attenuation of Aβ pathology and improved behavior [54], while another one with short-term IL-4 expression in mice exacerbated amyloid deposition [55]. The acute suppression of glial clearance activity due to the relative short duration of IL-4 exposure is a possible explanation to the inconsistency. IL-4 expression initiating time is another major difference of the two studies that worth further investigation. It worth mentioning that a IL-4 study has to be terminated prematurely due to the increased animal death after the intervention [56]. One possible interpretation for the death was the multiple cortex injection sites and resultant higher virus and cytokine load.

TGF-β, an immunosuppressive cytokine which protects neurons against damages, has a complex role in modulating Aβ pathology. Long-term overexpressing TGF-β by astrocytes in transgenic mice led to increased clearance of Aβ plaque by activated microglia [57] and improvement of Aβ-induced behavior impairment [58]. However, TGF-β can also promote astrocytes aggregating around brain microvessels and Aβ deposits on the vascular basement membranes [59–62]. Therefore, TGF-β can reduce Aβ pathology of brain parenchyma while at the same time cause the blood perfusion impairment in the associated regions.

IFN-γ is a pleiotropic cytokine which has a similar but weaker function to IL-4 in upregulating glial MHC class II [44], implying an immunosuppressive feature of the cytokine. The level change of IFN-γ in AD patients has not been reported, however, overexpressing IFN-γ results in a significant decrease of Aβ deposits and infiltration of peripheral monocytes [63], which is consistent to the observations that IFN-γ increases Aβ uptake by microglia and activates microglia to facilitate T cell motility and synapse formation in vitro [64].

The microglia-derived IL-12 and IL-23 is up-regulated in APP/PS1 transgenic mice and blocking these cytokines reverses the Aβ burden and the cognitive impairment [65]. Another study using accelerated senescence mice (SAMP8) reproduced the results [66]. In addition, a linear correlation of cognitive performance and CSF levels of p40, the common unit of IL-12 and IL-23, in AD subjects further supports the role of IL-12 and IL-23 in AD pathogenesis. IL-18, a member of IL-1 family, was elevated in LPS-stimulated blood mononuclear cells and brains of AD patients, and a significant correlation between IL-18 production and cognitive decline was observed [67, 68]. IL-18 promotes APP processing [69], tau phosphorylation [70] and can modulate the production of other cytokines [71]. Similarly, another IL-1 family member, IL-33 and its receptor ST2, showed strong expression in the AD brains, and incubation with Aβ increased astrocytic IL-33 expression [72]. The in vivo evidence of IL-18 and IL-33 in AD pathogenesis is currently missing and further studies may also explore whether these cytokines are detectable in CSF or serum of AD.

## Cytokines related to AD-like tau abnormalities

Abnormal post-translational modification of tau proteins plays a crucial role in AD neurodegeneration, and hyperphosphorylation is one of them that has been most extensively studied [73, 74]. Accumulating studies suggest that targeting the down-regulated protein phosphatase-2A (PP2A) [75, 76] or up-regulated glycogen synthase kinase-3β (GSK-3β) [77–80] or modulating the upstream membranous receptors may attenuate tau hyperphosphorylation [81, 82]. Currently, the role of tau in the neuroinflammation process of AD remains poorly understood and is far less studied compared to Aβ. However, the interplay between tau and cytokines has shed a light on the relevant mechanisms.

Pro-inflammatory cytokines have shown a consistent impact on tau pathology. Overexpression of IL-1β in 3xTg AD mice exacerbated tau hyperphosphorylation within one month [41], while blocking IL-1β signaling via IL-1 receptor antagonist (IL-1ra) or anti-IL-1β antibody reversed the cognitive impairment with a diminished tau pathology [83, 84]. The decreased activity of IL-1β-dependent tau kinases, such as cyclin-dependent kinase-5 (CDK5)/p25, GSK-3β and p38-mitogen activated protein kinase (MAPK) contributed to the reduction of phosphorylated tau [41, 83]. Additionally, a recent study showed that microglia can drive tau pathology, pathological tau spreading and memory impairment in the human tau40 mice through a IL-1β-dependent pathway since the inclusion of IL-1ra significantly reduced microglia-induced tau pathology [85]. 3, 6'-dithiothalidomide, a TNF-α-lowering agent, had no effect on total tau levels, but reduced phosphorylated tau in 3xTg AD mice [37]. Another study used a different TNF-α modulator, IDT in the same animal models also reduced paired helical filament tau (PHF-tau) and improved the cognition [39]. Treating hippocampal neurons with physiologic dose of IL-6 exhibited an increase in the amount of hyperphosphorylated tau of AD type, which may be attributed to an increased activity of CDK5/p35 complex [86].

In primary glial cultures, recombinant adeno-associated virus (rAAV)-mediated expression of IFN-γ did not alter endogenous tau production or phosphorylation. However, IFN-γ increased hyperphosphorylation and conformational changes of soluble tau in two animal models with tauopathy [87]. In turn, overexpressing tau40 increased secretion of TNF-α, IL-1β, IL-6, IL-10 and NO in rat microglia, which show greater phagocytosis of microspheres [88]. However, the phenotype of the microglia and how this phenotype would influence the Aβ pathology need further studies. Moreover, upregulating PP2A in astrocytes stimulates astrocytes migration via inhibiting p38-MAPK in Tg2576 mice [89], indicating that the tau-associated pathology may be involved in the impaired Aβ clearance.

It seems that tau pathology can be consequence of the deregulated inflammation, or serve as an inflammation promoter like Aβ to exacerbate inflammation. Nevertheless, to what extent tau may influence the inflammation, and what will be the sum effects of tau, Aβ and inflammation are mostly unknown. Besides, no related studies so far have examined the influence of anti-inflammatory cytokines on tau pathology. Cytokines also have important influence on neuron survival [90–93], blood brain barrier (BBB) integrity [94] and other normal physiological events in the CNS [3, 4], which cannot be reflected in animal models of single type of pathology. Thus, a more careful examination of the current animal models [95] and developing novel models more close to the real pathology of AD are needed [28].

## The adaptive immune system in AD

The most recent evidence has shown presence of a classical lymphatic system in the CNS [96], suggesting a frequent communication of the immune activities between periphery and the CNS on a regular basis. Over 80 % of the T cells in the CSF are CD4+ that can be classified into four subsets, including type 1 helper-inducer T (Th1) cells and Th17 cells defined as pro-inflammatory; and Th2 cells and regulatory T (Treg) cells defined as anti-inflammatory. The activating state and subtype of T cells in the circulation, CSF and parenchyma are modified in AD patients [97, 98]. In an immune-deficient AD mouse model, lack of T, B, and natural killer cells exhibits an increased Aβ with decreased phagocytic efficiency of microglia and significant elevation of several key pro-inflammatory cytokines including IL-1β, IL-6 and TNF-α [99]. These findings strongly suggest the active involvement of the adaptive immune system in AD pathogenesis.

Previous studies have highlighted the importance of cytokines in mediating the activity of peripheral immune cells in AD. Cytokines can facilitate the peripheral immune cells infiltration into the brain, resulting in direct Aβ phagocytosis by recruiting immune cells or inducing phagocytic activity of other cell types, such as microglia. The choroid plexus (CP) stroma is enriched with CD4+ T cells that are able to produce IL-4 and IFN-γ [98], and the IFN-γ plays an essential role in assisting leukocyte trafficking [100]. Decreased IFN-γ level in both 5XFAD and APP/PS1 mice were reversed by transient depletion of Treg cells at intermediate stage of AD, which at the same time led to increased leukocyte infiltration and recruitment to Aβ plaques, and attenuation of the AD pathology [101]. However, amplification of Treg cells at early disease stages through peripheral low-dose IL-2 treatment increased numbers of plaque-associated microglia, and restored cognitive functions in APP/PS1 mice [102]. Therefore, a more careful examination of Treg cells in different stages of the disease may help determining the proper therapeutic strategies.

Furthermore, when co-cultured with Aβ-treated microglia, the secretion of Th1 and Th17 cells increases, which then up-regulates MHC II, co-stimulatory molecules and pro-inflammatory cytokines in microglia [103, 104], thus improving the efficiency of presenting antigens to the T cells of microglia and enhancing Aβ clearance by both. However, IL-17 and IL-22, which are exclusively produced by Th17 cells, can also cause BBB disruption and infiltration of Th17 cells, but led to a direct injury to the neurons by Th17 cells via Fas/FasL pathway in Aβ-induced AD model rats [105]. In addition, respiratory infection of APP/PS1 mice increased infiltration of IFN-γ + and IL-17+ T cells into the brains of older mice and this was correlated with an increased Aβ level [106]. Together, these studies indicate that future studies should consider the complex interplay among many participants as seen in the real situation of AD.

The basal level of anti-inflammatory cytokines in CSF may help skewing the infiltrating T cells to the Th2 or Treg phenotype in physiological condition [98]. In AD patients, the pro-inflammatory cytokines in CSF increases, which induces more Th1 or Th17 cells that can be detrimental. Several in vivo studies via cerebral ventricles or systemic administration to examine the impact of cytokines or the relevant antibodies on AD pathology (see Table 2), the concomitant influence on the transformation of T cells phenotypes and following effects should be taken into consideration for a more reasonable interpretation of the outcomes.

## Cytokines as potential biomarkers for AD diagnosis

So far, a CSF signature of low Aβ_1-42_ and high tau concentrations and significant retention in PET imaging with amyloid tracers are suggested as the standard diagnostic criteria, with the highest specificity and accuracy [107]. However, lumbar puncture required for CSF has limited its application. Thus, novel biomarkers based on more accessible materials, such as plasma, are attractive in improving AD diagnosis. Several cytokines have shown disease progression-dependent manner, which suggests that cytokines may serve as potential disease predictors. For instance, data collected from a 20-years cohort study demonstrate greater possibility of cognitive impairment in individuals with increased IL-6 [108]. After reviewing 118 research articles and comparing 66 cytokines in plasma or CSF obtained from MCI and AD, it was found that the cytokines increased steadily or had peak level upon the transformation from MCI to AD. This may help predicting the risk of suffering from AD and recognizing AD subgroups, such as IL-1β, IL-6, TNF-α, IL-18, monocyte chemotactic protein (MCP)-1 and IL-10 [8]. However, in the latest meta-analysis, no significant differences in cytokines such as IL-1β, IL-6, IL-8, IL-10 or TNF-α were found between subjects with MCI and healthy controls, while significant heterogeneity was observed in some comparisons [109].

Considering the unstable outcome of single cytokine level, combinational use of multiple proteins is a more reasonable approach. However, since the first AD predicting model made up of 18 plasma biomarkers containing multiple cytokines has been proposed [110], few biomarker sets have shown stable performance and good reproducibility [111, 112]. Nevertheless, by using multiplex assays, two research groups have independently set up a panel of plasma proteins recently. These two panels are of high reproducibility and diagnostic accuracy, which were strongly associated with severity and progression of AD [113, 114]. Although no cytokines were involved in neither of the panels, one of the studies found positive correlations between the biomarkers and some cytokines altered in AD [114]. In addition, after screening 120 inflammatory molecules in CSF and serum of AD, MCI and healthy controls through protein-array analysis, a combination of soluble IL-6 receptor (sIL-6R), tissue inhibitor of metalloproteinases-1 (TIMP-1) and soluble TNF-α receptor I (sTNFR-I) in CSF was found to provide the best prediction to AD among other molecules [115].

Certainly, these results still need further verification by other research groups, while the heterogeneity in BBB integrity, physical state and disease stage of patients should be taken into consideration at the same time [8, 116]. Besides, the lack of standardization of sample collections or detections remains the dominant cause of failure of developing serum-based AD biomarkers. To address this problem, many organizations raise guidelines for standardization of blood-based biomarker studies in AD, covering the pre-blood draw, blood collecting, processing and storage [117]. Furthermore, longitudinal sampling over years [8] is a better approach to eliminate heterogeneity but needs optimization of its feasibility.

Although no evidence supports a direct association of systemic infections with AD [118–120], some specific pathogens have been identified as potential risks for AD, such as Herpes simplex virus type 1, *Chlamydophila pneumoniae*, *Helicobacter pylori* and periodontal bacteria [121]. A recent study shows that the infection burden (IB) consisting of common pathogens is associated with AD after adjusted for ApoE genotype and various comorbidities. AD patients or healthy controls with more seropositivities have significantly higher serum levels of IFN-γ, TNF-α and IL-6 [122]. As IB is a relatively stable indicator of systemic inflammation burden, the practical value of combinational use of IB with other biomarkers worth further investigations. Overall, single type of biomarker is far from enough to classify all phenotypes and stages of AD, the combination of plasma cytokines and other factor is the most realistic and promising approach to develop convenient and practical plasma biomarkers for AD.

## Cytokines as potential targets for AD therapy

The anti-inflammatory therapies using non-steroidal anti-inflammatory drugs (NSAIDs) were once considered promising. However, after the positive reports from the pioneering randomized trial of indomethacin [123, 124], the followed trials have not reached a definitive conclusion [28]. Lately, two meta-analyses have been conducted to reevaluate the role of NSAIDs in AD. Although it supported the use of NSAIDs for prevention of AD, there were no positive results from the randomized control trials (RCTs) [125, 126]. Moreover, in a follow-up evaluation study of the randomized AD anti-inflammatory prevention trial (ADAPT) and its follow-up study (ADAPT-FS) that treatment for 1 to 3 years with naproxen, a nonselective cyclooxygenase (COX) inhibitor, or celecoxib (a selective COX-2 inhibitor), the results show no prevention for the onset of dementia or no attenuation for the cognitive functions in older adults with a family history of AD [127]. Many reasons to the failure have been proposed, including duration of treatment [127], ApoE *ε4* allele [128, 129], ages [127], disease stages [130] and disease progressing speeds [131]. Therefore, long-term and large-scale RCTs based on more tolerable novel NSAIDs are needed for understanding the positive findings from molecular and epidemiologic studies. In the absence of such RCTs, indirect treatment comparisons or mixed treatment comparisons may also help to reach more robust conclusions [125].

As the broad anti-inflammatory medications are not promising, more specific immune pathways or molecules that are not affected by NSAIDs may be targeted. Etanercept is a TNF-α inhibitor originally used in the treatment of rheumatoid arthritis (RA). A noticeable clinical improvement was observed in AD patients minutes after perispinal administration of etanercept [132]. To explain the rapid effect of etanercept, the authors propose that the vertebral venous system may be an anatomical route to bypass the BBB and to deliver high molecular drugs to the CNS [133]. However, a recent study has challenged this claim, as three radio iodinated drugs including etanercept, were perispinally injected but the drug was not visualized in all but one of the rats using PET [134]. Recent studies indicate that intravenously-administered etanercept has no apparent clinical benefit to AD patients, although good tolerability of subcutaneous etanercept over a 24-week period was observed [133, 135], suggesting better effects by perispinal administrating compared to peripheral route. Together, these studies confirm the pathogenic role of TNF-α in AD and show great potential of anti-TNF-α therapies through various administration routes.

Although targeting cytokines is a relatively new approach compared to other anti-inflammatory therapies in AD, it is noteworthy that a great number of cytokine inhibitors have already been successfully used in the treatments of autoimmune diseases and cancers [136, 137], and more biologics are under development [138]. Repurposing these drugs in AD treatments could be a reasonable approach. For instance, IL-1ra is decreased in CSF of AD patients [139] and its protecting effect towards AD has been confirmed in animal models [84, 85]. Although there is still no clinical evidence supporting the use of IL-1ra in AD patients, the success in treating RA and cortical infarcts [140] makes it a very promising target in AD treatments. Similarly, p40-neutralizing antibodies, which block the IL-12/IL-23 signaling pathway, have been approved by Food and Drug Administration (FDA) for the treatment of psoriasis, thus may be ideal for the initiation of clinical trials [65].

Besides, indirect approaches such as targeting upstream regulators of the cytokine expression seem also attractive. For instance, the Aβ-dependent induction of IL-1β requires two sequential signals. The first signal is triggered by Aβ binding to the toll-like receptors (TLRs) and leads to the production of IL-1β precursor. The second signal occurs via NLRP3 (NACHT, LRR and PYD domains-containing protein 3) inflammasome activation, which requires cathepsin B leakage from phagolysosomes or mitochondrial damage, and the subsequent reactive oxygen species (ROS) production. Then the NLRP3 inflammasome can activate caspase-1, which processes the pro-IL-1β into its bioactive form [141]. Although there are no FDA-approved drugs that exclusively and specifically target NLRP3, a small molecule inhibitor of NLRP3 has been identified [142]. Therefore, more initiative attempts of repurposing anti-cytokine drugs in AD treatments and more careful assessments of the results may lead to unexpected cheerful outcomes.

## Conclusions

The cytokines are involved in various physiological and pathological pathways, therefore, inconsistent results have been observed in AD pathologies and treatment. The present evidence strongly indicates that dysregulation of the cytokines drives pathogenic process primarily through influencing the phenotype of microglia, and co-existence of both pro-inflammatory cytokines and the suppressing state of microglia may represent an irreversible point of the disease. Future studies on AD should extend to more pathogens than Aβ, and investigate the interplay between cytokine and other participators. The genome-wide association studies and the online database analysis will provide continuously updated polymorphism information associated with AD, while development of brain banks is critical for identification of new genes and proteins [143]. Given that increasing studies have proven the role of adaptive immune system in AD, the impact of peripheral T cells and relevant cytokines cannot be ignored in future studies. As the immune events may change during the disease course and the heterogeneity in AD, it is not necessarily that all individuals with AD exhibit neuroinflammation, or at all-time points in the course of the disease. To learn from the existing therapy strategies of other related inflammatory diseases or to develop novel cytokine inhibitors could be reasonable approaches to making progress in AD anti-inflammatory therapies.

## Abbreviations

AD: Alzheimer’s disease
ADAPT: anti-inflammatory prevention trial
ADAPT-FS: anti-inflammatory prevention trial-follow-up study
ApoE: apolipoprotein E
APP: amyloid precursor protein
Aβ: beta amyloid
BBB: blood brain barrier
CDK5: cyclin-dependent kinase-5
CNS: central nervous system
COX: cyclooxygenase
CP: choroid plexus
CSF: cerebral spinal fluid
FDA: food and drug administration
GSK: glycogen synthase kinase
htau40: human tau40 protein
IDE: insulin-degrading enzyme
IFNs: interferons
IGF-1: insulin-like growth factor-1
IL-1ra: IL-1 receptor antagonist
ILs: interleukins
MAPK: mitogen activated protein kinase
MCI: mild cognitive impairment
MCP-1: monocyte chemotactic protein -1
MHC-II: major histocompatibility complex class II
NFTs: neurofibrillary tangles
NSAIDs: non-steroidal anti-inflammatory drugs
PHF-tau: paired helical filament tau
PP2A: protein phosphatase-2A
PSEN: presenelin
RA: rheumatoid arthritis
rAAV: recombinant adeno associated virus
ROS: reactive oxygen species
SAMP8: senescence accelerated mouse
sIL-6R: soluble IL-6 receptor
sTNFR-I: soluble TNF-α receptor I
TGFs: transforming growth factors
Th1 cells: type 1 helper-inducer T cells
TIMP-1: tissue inhibitor of metalloproteinases-1
TLRs: toll-like receptors
TNFs: tumor necrosis factors
Treg cells: regulatory T cells
TREM2: triggering receptor expressed on myeloid cells 2
α1-ACT: α1-antichymotrypsin
